# Interplay between cost and effectiveness in influenza vaccine uptake: a vaccination game approach

**DOI:** 10.1098/rspa.2019.0608

**Published:** 2019-12-18

**Authors:** Md. Rajib Arefin, Tanaka Masaki, K. M. Ariful Kabir, Jun Tanimoto

**Affiliations:** 1 Interdisciplinary Graduate School of Engineering Sciences, Kyushu University, Kasuga-koen, Kasuga-shi, Fukuoka 816-8580, Japan; 2 Faculty of Engineering Sciences, Kyushu University, Kasuga-koen, Kasuga-shi, Fukuoka 816-8580, Japan; 3 Department of Mathematics, University of Dhaka, Dhaka-1000, Bangladesh; 4 Department of Mathematics, Bangladesh University of Engineering and Technology, Dhaka, Bangladesh

**Keywords:** vaccination game, epidemic model, influenza vaccine

## Abstract

Pre-emptive vaccination is regarded as one of the most protective measures to control influenza outbreak. There are mainly two types of influenza viruses—influenza A and B with several subtypes—that are commonly found to circulate among humans. The traditional trivalent (TIV) flu vaccine targets two strains of influenza A and one strain of influenza B. The quadrivalent (QIV) vaccine targets one extra B virus strain that ensures better protection against influenza; however, the use of QIV vaccine can be costly, hence impose an extra financial burden to society. This scenario might create a dilemma in choosing vaccine types at the individual level. This article endeavours to explain such a dilemma through the framework of a vaccination game, where individuals can opt for one of the three options: choose either of QIV or TIV vaccine or none. Our approach presumes a mean-field framework of a vaccination game in an infinite and well-mixed population, entangling the disease spreading process of influenza with the coevolution of two types of vaccination decision-making processes taking place before an epidemic season. We conduct a series of numerical simulations as an attempt to illustrate different scenarios. The framework has been validated by the so-called multi-agent simulation (MAS) approach.

## 1. Introduction

Seasonal influenza costs lots of lives throughout the world every year, causing a huge economic burden to society, involving treatment costs and productivity losses. Costs due to seasonal influenza have been reported as 2–6% of gross domestic product (GDP) *per capita* in low- and middle-income countries (LMICs), compared to only 0.04–0.13% of GDP in developed or high-income countries [1,2]. There are four main types of influenza viruses, A, B, C and D, that have been identified so far [3], among which influenza A and B viruses are the most common among humans that cause seasonal epidemics worldwide; however, circulation patterns and strain prevalence can be region-specific and time-specific [4]. According to a report published by the Centers for Disease Control and Prevention (CDC) [5], the United States 2017–2018 influenza season (1 October 2017 to 19 May 2018) faced severity from both influenza viruses; influenza A viruses prevailed at the beginning of the season but influenza B predominated in the later part of the season. As reported by previous studies, influenza A viruses have two strain subtypes, H1N1 and H3N2, influenza B viruses have two subtypes—B/Victoria and B/Yamagata—that have been commonly found to circulate [6,7].

Annual vaccination against influenza is suggested to be the most effective strategy to protect from the influenza disease. The traditional trivalent (TIV) vaccine targets three strain subtypes of influenza viruses (two strains of A and one of the B virus strains); on the other hand, the quadrivalent (QIV) vaccine contains one extra strain of influenza B viruse that offers improved protection against influenza B virus infections [3]. Each year public health authorities recommend which strain should be contained in vaccines for the upcoming season (for example [5,8] for USA). However, due to the mismatch between the recommended B virus strain for TIV vaccine and the circulating virus strain, influenza morbidity may increase [4,9]. Inclusion of an extra B strain to QIV vaccine provides better efficacy than that of TIV vaccine. Because they provide better protection, some of the developed countries have been turning towards QIV vaccines, although TIV vaccines are still recommended [8]. Nonetheless, many other countries including Russia are still relying on TIV vaccine [4]. Moreover, the use of QIV vaccine can be substantially expensive compared to TIV and may impose a considerable economic burden on LMICs, although the long-term impact of implementing QIV can be cost-effective [2,10].

So far, a significant amount of research has been conducted investigating the cost-effectiveness of TIV and QIV vaccine usages for developed countries and LMICs [2,10–13]. Most of these studies used an individual-based dynamic simulation model (4flu) using demographic data to evaluate the impact of TIV and QIV vaccines. However, in addition to those analyses, we also need to ponder individualistic assessment on choosing vaccine types (when both vaccines are available) as vaccination is usually voluntary in a social perspective. Thus, we need to consider individual perception on taking the vaccination with disease spreading to have a better understanding of the immunization programme. Here, we attempt to analyse people's vaccination decision (TIV or QIV or none) depending upon cost, effectiveness and other factors in the framework of a vaccination game [14–30] and then illustrate the resulting impact in a societal perspective. The vaccination game allows a framework that elucidates behaviour–disease–vaccination interactions with the help of evolutionary game theory [31–34], which has been used to study the conflict between self-interest and social interest in various circumstances [35–38]. Individuals who commit to vaccination (cooperators) can benefit themselves as well as their surroundings; however, people who do not vaccinate (defectors), expose themselves to the risk of infection that may cause illness and more financial burden compared to the vaccination cost, or they may become free riders, benefiting from ‘herd immunity’ that can be attained from the situation when a larger portion of the population is vaccinated. This scenario creates a social dilemma that can be explained by the vaccination game [35,39]. In this work, we design a mean-field vaccination game in an infinite and well-mixed population, with three strategies: taking QIV vaccine, TIV vaccine, and neither QIV nor TIV. Our approach follows a similar avenue to that of Kuga & Tanimoto [16], where the vaccination is assumed to be pre-emptive. Notably, the authors in [16] successfully developed an equivalent mean-field framework of a vaccination game, taking inspiration from [40], which mainly focused on a networked population. A similar work related to [40] can be found in [41]. The number of vaccinees remains the same during an epidemic season as we are considering seasonal influenza. Moreover, as influenza vaccines are not perfectly effective [42], we take into account vaccine efficacy in our modelling. The assumption of imperfect vaccine in the vaccination game can tackle more realistic situations, and lead to interesting results, which otherwise can be intractable (for instance, see [43,44]). In our current setting, at the end of each season, individuals assess their perceived payoffs based on the previous season's experience in terms of vaccine efficacy, cost, etc., and decide whether to vaccinate or not or if vaccinating then which vaccine to take for the next season. The strategy adaptation after each season is based on imitating others' strategy with better payoffs. The group who chooses QIV vaccine are expected to get better protection against the disease than that of the group taking TIV vaccine, although this may depend on the circulation of influenza B virus. However, the cost for TIV vaccine should be no more than the cost for QIV vaccine. Taking into account all these factors, we combine the disease spreading dynamics of influenza with the vaccination decision-making process through an evolutionary framework. We perform our simulations for repeated seasons until we reach a social equilibrium (steady state). By varying vaccine effectiveness and cost level, our numerical simulations intend to show an overall picture of influenza epidemics. At equilibrium, we calculate to what extent the average social payoff attained from the vaccination game falls short from the desired or social optimum payoff, which we name as, ‘social efficiency deficit’. Moreover, our approach has been validated with the multi-agent simulation (MAS) approach. We also explore the robustness of our framework in explaining the situation with influenza A or B virus-dominant circulation.

The rest of this paper is organized as follows: §2 provides the detailed illustration of the model covering the epidemic spreading, payoff structure, strategy update and the evolutionary dynamics of vaccination coverage. Section 3 delivers a comprehensive discussion resulting from a series of numerical simulations. Section 4 concludes the findings of this work and provides some future indications.

## 2. Model description

**Figure 1.**
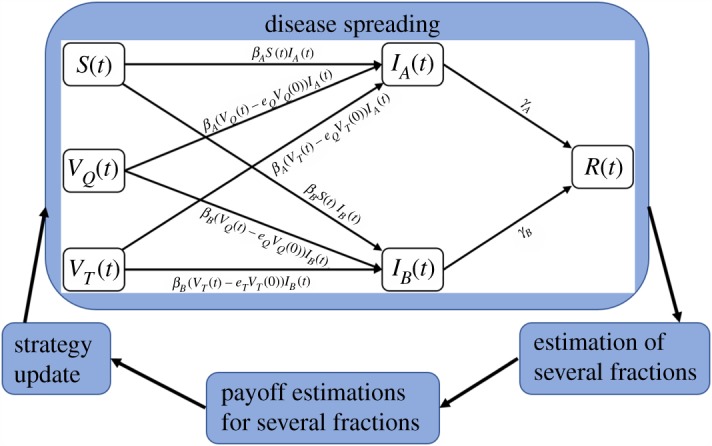
The layout of the whole dynamical set-up. The vaccine efficacy of TIV vaccine against influenza B virus is assumed *e_T_*, which is lower than that of QIV vaccine. However, both vaccines are assumed to have same efficacy (*e_Q_*) against influenza A virus. Once the disease spreading process ends, we estimate several fractions such as vaccinated and healthy, vaccinated but infected, infected with influenza A(B), etc., and evaluate their payoffs. These fractions then update their strategies for the next season. This process is repeated until we reach a steady state. Arrows depict the sequence of the evolutionary process. (Online version in colour.)

We entangle the simultaneous spreading of two influenza viruses (A and B) and the evolution of vaccination (QIV or TIV or none) decision by constructing a repetitive sequence of a two-stage process in an infinite and well-mixed population. The disease spreading process is governed by susceptible-infected-recovered (SIR)-like dynamics [45] coupled with vaccination that allow us to estimate several fractions of individuals at the end of each epidemic period. These different groups then evaluate their payoffs and revisit their vaccination strategies by copying other individuals' strategy who achieved higher payoffs in the previous season. Finally, we update the fraction of vaccinees of both types (QIV and TIV) using evolutionary equations given in §2e. Let us note that the whole process is repeated for several steps (generations) until we reach a steady state. Figure 1 illustrates the whole dynamical set-up.

### (a) Epidemic spreading

The first stage of our dynamical system is the disease spreading process that is based on a SIR-like model coupled with vaccination. Initially, we divide the whole population into three categories: susceptible (unvaccinated) denoted by *S*, QIV vaccinees denoted by *V_Q_* and TIV vaccinees denoted by *V_T_*. It is not usually recommended to take both vaccines (TIV and QIV) in a single season [46]; therefore, we disregard any group taking both vaccines in the same season. As influenza vaccines are not 100% perfect, we consider imperfect vaccinations in our disease modelling, where both vaccines are presumed to provide the same level of efficiency against A virus but different efficacies against B virus. The inclusion of these parameters allows us to consider some individuals from vaccinated groups who fail to get immunity from vaccination (QIV or TIV) and still face the risk of infection like susceptible. The unvaccinated or vaccinated people who fail to get immunity may become infected with either influenza virus (A or B), and then these infected people transfer to the recovery state after suffering from illness. Therefore, at time *t*, an individual can be at any of the six compartments: susceptible, *S*(*t*); QIV vaccinees, *V_Q_*(*t*); TIV vaccinees, *V_T_*(*t*); infected with influenza A, *I_A_*(*t*); infected with influenza B, *I_B_*(*t*); recovered state, *R*(*t*). We presume *e_Q_* (0 ≤ *e_Q _*≤ 1) as the vaccine efficacy of QIV against both influenza A and B viruses. As TIV (QIV) vaccine contains two strains of influenza A virus and one (two) strain(s) of influenza B virus, we assume that both QIV and TIV vaccines bestow the same level of effectiveness (*e_Q_* (0 ≤ *e_Q_* ≤ 1)) against influenza A but TIV vaccine provides relatively lower effectiveness, *e_T_* (0 ≤ *e_T_* ≤ *e_Q_*) against influenza B than that of QIV vaccine. If *V_Q_*(0) is the fraction of vaccinees at the beginning of an epidemic season, then a proportion *e_Q_* of *V_Q_*(0) i.e. *e_Q_V_Q_*(0) gets perfect immunity from QIV vaccine and, the remaining fraction, *V_Q_*(*t*) − *e_Q_V_Q_*(0) becomes vulnerable to the disease, where *V_Q_*(*t*) is the fraction of vaccinators in the *V_Q_* compartment at time *t*. A similar logic has been used for TIV vaccinees. The transmission rates and recovery rates for influenza A and B viruses are assumed *β_A_*, *γ_A_* and *β_B_*, *γ_B_*, respectively. It is worth mentioning that the state space variables represent several fractions of the total population; therefore, we are not considering a population of any particular size. Although coinfection and superinfection with two influenza viruses are possible in some cases [47,48], the incidence rate is not so significant. Therefore, our model disregards the incidence of coinfection and superinfection with two influenza viruses (A and B) and presumes long-term cross-immunity between them. Although we assume long-term cross-immunity between two viruses, short-term immunity is possible in reality. The incidence of short-term cross-immunity between two viruses may allow some individuals recovered from one virus to be infected with the other one after a short time interval (e.g. [49]). The schematic of our epidemic model is given in the disease spreading part of figure 1. The model equations are given by

2.1 $$\begin{array}{rl} \frac{\text{d}S(t)}{\text{d}t} & =-\beta_{A}S(t)I_{A}(t)-\beta_{B}S(t)I_{B}(t),\\ \frac{\text{d}V_{Q}(t)}{\text{d}t} & =-\beta_{A}(V_{Q}(t)-e_{Q}V_{Q}(0))I_{A}(t)-\beta_{B}(V_{Q}(t)-e_{Q}V_{Q}(0))I_{B}(t),\\ \frac{\text{d}V_{T}(t)}{\text{d}t} & =-\beta_{A}(V_{T}(t)-e_{Q}V_{T}(0))I_{A}(t)-\beta_{B}(V_{T}(t)-e_{T}V_{T}(0))I_{B}(t),\\ \frac{\text{d}I_{A}(t)}{\text{d}t} & =\beta_{A}S(t)I_{A}(t)+\beta_{A}(V_{Q}(t)-e_{Q}V_{Q}(0))I_{A}(t)+\beta_{A}(V_{T}(t)-e_{Q}V_{T}(0))I_{A}(t)-\gamma_{A}I_{A}(t),\\ \frac{\text{d}I_{B}(t)}{\text{d}t} & =\beta_{B}S(t)I_{B}(t)+\beta_{B}(V_{Q}(t)-e_{Q}V_{Q}(0))I_{B}(t)+\beta_{B}(V_{T}(t)-e_{T}V_{T}(0))I_{B}(t)-\gamma_{B}I_{B}(t)\\ \;\text{and}\;\frac{\text{d}R(t)}{\text{d}t} & =\gamma_{A}I_{A}(t)+\gamma_{B}I_{B}(t). \end{array}\}$$

It is obvious that *S*(*t*) + *V_Q_*(*t*) + *V_T_*(*t*) + *I_A_*(*t*) + *I_B_*(*t*) + *R*(*t*) = 1. If *x*, *y *∈ [0, 1] (*s.t.* *x* + *y* ≤ 1) are the fractions of QIV and TIV vaccinees at the beginning of a season, i.e. *V_Q_*(0) = *x*, *V_T_*(0) = *y*, then *S*(0) = 1 − *x* − *y* − *ε_A_* − *ε_B_*, where *ε_A_*( → 0^+^) and *ε_B_*( → 0^+^) are initial infections with influenza A and influenza B, respectively.

### (b) Outcome of disease spreading

After each epidemic season, we estimate several groups by numerically calculating fluxes from one state to another. We mainly estimate nine fractions of individuals that are, QIV vaccinees and healthy-HV*_Q_*, QIV vaccinees but infected with influenza A(B) virus-*V_Q_I_A_* (*V_Q_I_B_*), TIV vaccinees and healthy-HV*_T_*, TIV vaccinees but infected with influenza A(B) virus-*V_T_I_A_* (*V_T_I_B_*), successful free riders-SFR (who are unvaccinated but did not suffer from any influenza virus), failed free riders-FFR*_A_* or FFR*_B_* (unvaccinated individuals infected by either influenza virus). Individuals who remain at *S*, *V_Q_* and *V_T_* state at equilibrium (*t* → ∞), that is *S*(∞), *V_Q_*(∞) and *V_T_*(∞), are termed successful free riders, healthy QIV vaccinees and healthy TIV vaccinees, respectively. Suppose φ*_P_*_→_*_Q_* is the total proportion transferred from state *P* to state *Q* during an epidemic season. Thus, QIV vaccinees who get infected with influenza A(B) virus can be evaluated from the flux, $\varphi_{V_{Q}\to I_{A}}$ ($\varphi_{V_{Q}\to I_{B}}$). In a similar way, we can estimate this for TIV vaccinees. The failed free riders can be estimated by fluxes, $\varphi_{S\to I_{A}}$ and $\varphi_{S\to I_{B}}$.

### (c) Payoff structure

Once an epidemic season ends, several groups estimate their payoffs prior to the onset of the next epidemic season. Suppose the cost of vaccination and infection are, respectively, *C_V_* and *C_I_*. The vaccination cost includes the vaccine price with possible side effects due to vaccination. Since the infection cost is higher than the vaccination cost, we define the relative cost of vaccination as *C_r_* = *C_V_*/*C_I_*, (0 ≤ *C_r_* ≤ 1). Without loss of generality, we can choose *C_I_* = 1. Then the relative cost for QIV (TIV) vaccine can be assumed as *C_Q_*(*C_T_*). Since the cost for TIV is assumed to be no more than QIV, we can presume 0 ≤ *C_Q_* ≤ 1 and 0 ≤ *C_T_* ≤ *C_Q_*. Therefore, QIV(TIV) vaccinees who remain healthy during the epidemic period can have a payoff, − *C_Q_*(−*C_T_*); however, QIV(TIV) vaccinees who become infected with either influenza virus (A or B) have a payoff, −*C_Q_* − 1(−*C_T_* − 1). In addition, payoffs for successful free riders and failed free riders are assigned as 0 and −1, respectively. All fractions along with their payoffs are summarized in table 1.

**Table 1.** Several fractions of individuals (at equilibrium) with their payoffs (within brackets).

strategy/state	healthy	infected with influenza A	infected with influenza B
QIV vaccinees (*V_Q_*)	HV*_Q_* (− *C_Q_*)	*V_Q_I_A_* (− *C_Q_* − 1)	*V_Q_I_B_* (− *C_Q_* − 1)
TIV vaccinees (*V_T_*)	HV*_T_* (− *C_T_*)	*V_T_I_A_* (− *C_T_* − 1)	*V_T_I_B_* (− *C_T_* − 1)
non-vaccinated (NV)	SFR (0)	FFR*_A_* (− 1)	FFR*_B_* (− 1)

Using table 1, we estimate the average social payoff 〈*π*〉; average payoff for QIV vaccinees $\left(\langle \pi_{V_{Q}}\rangle \right)$, TIV vaccinees $\left(\langle \pi_{V_{T}}\rangle \right)$ and non-vaccinators (〈*π*_NV_〉) as follows:

2.2 $$\begin{array}{rl} \left\langle \pi \right\rangle & =-C_{Q}\text{H}{\text{V}}_{Q}+(-C_{Q}-1)(V_{Q}I_{A}+V_{Q}I_{B})-C_{T}\text{H}{\text{V}}_{T}+(-C_{T}-1)(V_{T}I_{A}+V_{T}I_{B})\\ & \;-(\text{FF}{\text{R}}_{A}+\text{FF}{\text{R}}_{B}), \end{array}$$

2.3 $$\begin{array}{rl} \left\langle \pi_{V_{Q}}\right\rangle & =\frac{-C_{Q}\text{H}{\text{V}}_{Q}+(-C_{Q}-1)(V_{Q}I_{A}+V_{Q}I_{B})}{x}, \end{array}$$

2.4 $$\begin{array}{rl} \left\langle \pi_{V_{T}}\right\rangle & =\frac{-C_{T}\text{H}{\text{V}}_{T}+(-C_{T}-1)(V_{T}I_{A}+V_{T}I_{B})}{y}, \end{array}$$

2.5 $$\begin{array}{rl} \;\text{and}\;\langle \pi_{\text{NV}}\rangle & =\frac{-(\text{FF}{\text{R}}_{A}+\text{FF}{\text{R}}_{B})}{1-x-y}. \end{array}$$

### (d) Strategy update

Individuals assess their payoffs after an epidemic season and decide whether to imitate others' strategy or stay with their previous season's strategy. In a seminal work regarding the agent-based vaccination game model, Fu *et al*. [40] first used the pairwise Fermi function [50,51] to calculate the likelihood of copying others' strategy by comparing payoffs, where a focal agent *i* having the strategy *S_i_* with the payoff *π_i_* adopts the strategy *S_j_* of a randomly chosen agent *j* having the payoff *π_j_* with the probability

2.6 $$P(S_{i}\leftarrow S_{j})=\frac{1}{1+\exp[-(\pi_{j}-\pi_{i})/k]},$$

where the parameter, *k *> 0 measures the strength of selection; smaller *k* signifies the strong selection, that is players are more responsive to the payoff difference. A typical choice of *k* is 0.1, which has been used in many previous studies such as [16,18–20,29]. Fukuda *et al*. [18] later slightly modified the formula (2.6), where rather than comparing with a single agent, a focal player *i* compares its payoff *π_i_* with an average payoff 〈*π_j_*〉 of all neighbours having the same strategy *S_j_* of a randomly selected neighbour *j*. The modified formula becomes

2.7 $$P(S_{i}\leftarrow S_{j})=\frac{1}{1+\exp\left[-\left(\langle \pi_{j}\rangle -\pi_{i}\right)/k\right]}.$$

Although formulae (2.6) and (2.7) have been mainly used for agent-based modelling, these can also be used for the mean-field approach (such as [16,20,28,52]). Inspired by that, our approach uses formula (2.7) to update vaccination strategies for the next season. In our case, every fraction (each of nine fractions in table 1) compares its payoff with the average payoff of a mutually exclusive group (QIV, TIV, non-vaccinated in table 1); for instance, the fraction, HV*_Q_* can, respectively, compare its payoff with the average payoff of TIV vaccinees group (*V_T_*) and non-vaccinators group (NV) by the formula

$$\begin{array}{rl} & P(\text{H}{\text{V}}_{Q}\leftarrow V_{T})=\frac{1}{1+\exp\left[-\left(\left\langle \pi_{V_{T}}\rangle -(-C_{Q}\right)\right)/k\right]}\\ \;\text{and}\; & P(\text{H}{\text{V}}_{Q}\leftarrow \text{NV})=\frac{1}{1+\exp\left[-\left(\left\langle \pi_{\text{NV}}\rangle -(-C_{Q}\right)\right)/k\right]}. \end{array}$$

Similarly, we estimate all necessary transition probabilities.

### (e) Evolutionary dynamics

As mentioned before, individuals update their vaccination strategies prior to onset of the next epidemic season. The evolution of vaccination strategies (QIV or TIV or unvaccinated) is estimated using the following evolutionary equations that have been derived by extending the master equation of the mean-field framework in a well-mixed population [52,53]

2.8 $$\begin{array}{rl} \frac{\text{d}x}{\text{d}t} & =-\text{H}{\text{V}}_{Q}.V_{T}.P(\text{H}{\text{V}}_{Q}\leftarrow V_{T})-V_{Q}I_{A}.V_{T}.P(V_{Q}I_{A}\leftarrow V_{T})-V_{Q}I_{B}.V_{T}.P(V_{Q}I_{B}\leftarrow V_{T})\\ & \;-\text{H}{\text{V}}_{Q}.\text{NV}.P(\text{H}{\text{V}}_{Q}\leftarrow \text{NV})-V_{Q}I_{A}.\text{NV}.P(V_{Q}I_{A}\leftarrow \text{NV})-V_{Q}I_{B}.\text{NV}.P(V_{Q}I_{B}\leftarrow \text{NV})\\ & \;+\text{H}{\text{V}}_{T}.V_{Q}.P(\text{H}{\text{V}}_{T}\leftarrow V_{Q})+V_{T}I_{A}.V_{Q}.P(V_{T}I_{A}\leftarrow V_{Q})+V_{T}I_{B}.V_{Q}.P(V_{T}I_{B}\leftarrow V_{Q})\\ & \;+\text{SFR}.V_{Q}.P(\text{SFR}\leftarrow V_{Q})+\text{FF}{\text{R}}_{A}.V_{Q}.P(\text{FF}{\text{R}}_{A}\leftarrow V_{Q})+\text{FF}{\text{R}}_{B}.V_{Q}.P(\text{FF}{\text{R}}_{B}\leftarrow V_{Q}) \end{array}$$

and

2.9 $$\begin{array}{rl} \frac{\text{d}y}{\text{d}t} & =-\text{H}{\text{V}}_{T}.V_{Q}.P(\text{H}{\text{V}}_{T}\leftarrow V_{Q})-V_{T}I_{A}.V_{Q}.P(V_{T}I_{A}\leftarrow V_{Q})-V_{T}I_{B}.V_{Q}.P(V_{T}I_{B}\leftarrow V_{Q})\\ & \;-\text{H}{\text{V}}_{T}.\text{NV}.P(\text{H}{\text{V}}_{T}\leftarrow \text{NV})-V_{T}I_{A}.\text{NV}.P(V_{T}I_{A}\leftarrow \text{NV})-V_{T}I_{B}.\text{NV}.P(V_{T}I_{B}\leftarrow \text{NV})\\ & \;+\text{H}{\text{V}}_{Q}.V_{T}.P(\text{H}{\text{V}}_{Q}\leftarrow V_{T})+V_{Q}I_{A}.V_{T}.P(V_{Q}I_{A}\leftarrow V_{T})+V_{Q}I_{B}.V_{T}.P(V_{Q}I_{B}\leftarrow V_{T})\\ & \;+\text{SFR}.V_{T}.P(\text{SFR}\leftarrow V_{T})+\text{FF}{\text{R}}_{A}.V_{T}.P(\text{FF}{\text{R}}_{A}\leftarrow V_{T})+\text{FF}{\text{R}}_{B}.V_{T}.P(\text{FF}{\text{R}}_{B}\leftarrow V_{T}). \end{array}$$

Here additions and subtractions, respectively, indicate inflows to and outflows from a state. We solve equations (2.8) and (2.9) numerically, allowing the system to reach the steady state as, *t* → ∞.

## 3. Results and discussion

This section deliberately explains results of the whole dynamical process. We vary several parameters such as transmission rates, vaccine effectiveness, vaccination cost, etc., to illustrate different scenarios. The symptoms of usual influenza sickness resolve after a period of 3–7 days, so the average durability in the infected stage is 5 days [49]. Therefore, we presume *γ_A_* = *γ_B_* = 1/5 = 0.2 throughout our numerical experiments.

### (a) Single season time series

**Figure 2.**
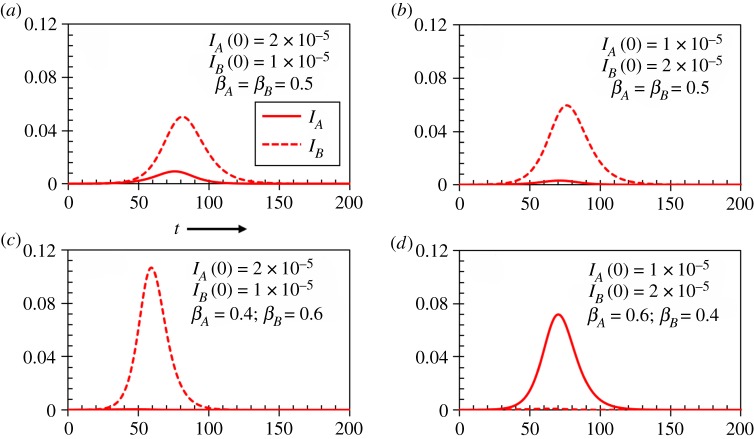
Time series of the fractions of infection by two influenza viruses (*A* and *B*) for different initial conditions and transmission rates without considering game approach. The vaccination coverage for each vaccine is assumed approximately 33% with vaccine effectiveness *e_Q_* = 0.6 and *e_T_* = 0.4. The recovery rate for each flu virus is chosen as 0.2. Under the current set-up, when *β_A_* = *β_B_* (*a*,*b*), B virus is found to dominate because *e_T_* ≤ *e_Q_*, whereas the flu virus with higher transmission rate (*c*,*d*) dominates the other one. The transmission rate seems more influential in virus dominance than the degree of initial dominance of each virus infection. (Online version in colour.)

Let us first briefly discuss the relative dynamics of infections due to influenza A and B viruses for different transmission rates and degree of initial infections in a single season without considering game aspect (figure 2). The vaccination coverage for this case is chosen as *V_Q_*(0) = *V_T_*(0) = 0.333 with *e_Q_* = 0.6, *e_T_* = 0.4. As *e_T_* ≤ *e_Q_*, influenza B virus seems to dominate in the case of equal transmission rate (*β_A_* = *β_B_*) (figure 2*a*,*b*)). However, the virus with higher transmission rate is found to dominate irrespective to the initial infection. Hence, the sensitivity of initial infections seems insignificant comparing to the transmission rates, i.e. transmission rates show more impact on virus dominance.

### (b) Evolutionary outcome of vaccination coverage

**Figure 3.**
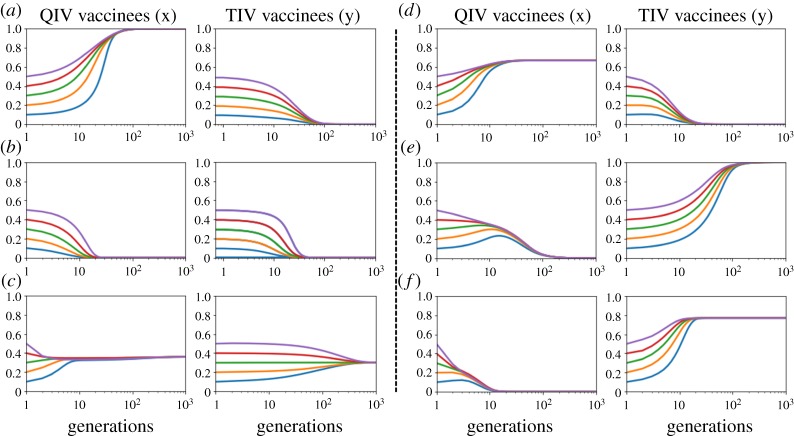
Six possible combinations of QIV and TIV vaccination coverages at equilibrium attained from the evolutionary equations (2.8) and (2.9). The evolutionary dynamics of the vaccination coverage depends upon the choice of different parameters. Clearly, there is no bi-stability at equilibrium. There are six possible mixtures of dynamics: (*a*) *x* = 1, *y* = 0, when *C_Q_* = *C_T_* = 0.4; *e_Q_* = 0.4, *e_T_* = 0.3. (*b*) *x* = 0, *y* = 0, when *C_Q_* = 0.6, *C_T_* = 0.4; *e_Q_* = 0.4, *e_T_* = 0.3. (*c*) *x* = x*, *y* = y*, (coexistence or internal equilibria) when *C_Q_* = 0.6, *C_T_* = 0.4; *e_Q_* = 0.8, *e_T_* = 0.4. (*d*) *x* = x* (internal equilibrium), *y* = 0, when *C_Q_* = *C_T_* = 0.4; *e_Q_* = 0.7, *e_T_* = 0.4. (*e*) *x* = 0, *y* = 1, when *C_Q_* = 0.6, *C_T_* = 0.4; *e_Q_* = 0.6, *e_T_* = 0.4. (*f*) *x* = 0, *y* = y*, when *C_Q_* = 0.6, *C_T_* = 0.4; *e_Q_* = 0.8, *e_T_* = 0.6. All cases presume, *β_A_* = *β_B_* = 0.5; *γ_A_* = *γ_B_* = 0.2. The initial value of *y* is kept as, 0.33, while we vary the initial values of *x* and vice versa. (Online version in colour.)

We estimate the coevolution of both types of vaccination at the end of each season. Owing to the complexity of equations (2.8) and (2.9), it is difficult to theoretically derive all possible equilibria; however, it is still possible to derive that numerically by tuning different parameters. Considering equal transmission rate for both viruses, we estimate six possible combinations of evolutionary outcomes (figure 3), which are (*x*, *y*) ≡ (1, 0);(*x*, *y*) ≡ (0, 0);(*x*, *y*) ≡ (x*, y*);(*x*, *y*) ≡ (x*, 0);(*x*, *y*) ≡ (0, 1);(*x*, *y*) ≡ (0, y*); where 0 < x*, y* < 1. Figure 3 suggests that there is none of the bi-stability arising from equations (2.8) and (2.9). One possible reason is that the present model as a nonlinear dynamical system instinctively has strong sinks, or steeply curved orbits, being insensible to initial conditions. Let us note that we choose initial values of *x* and *y* in such a way so that *x* + *y* ≤ 1. Moreover, when we vary initial values of *x*, the initial value of *y* is set as 0.33 and vice versa.

### (c) Phase plane analysis

#### (i) Case I (*β_A_* = *β_B_*)

**Figure 4.**
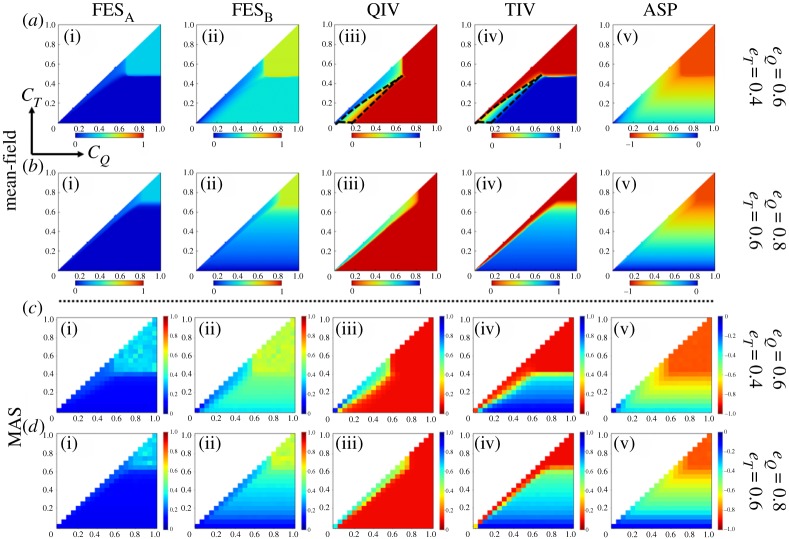
*C_Q_* versus *C_T_* (*C_T_* ≤ *C_Q_*) 2D-heatmaps with different effectiveness levels presuming mean-field (with *β_A_* = *β_B_* = 0.5, *I_A_*(0) = 0.00001, *I_B_*(0) = 0.00002) and multi-agent simulation (MAS) approach (in well-mixed population). Chronologically each column represents the final epidemic size for influenza A (FES*_A_*), the final epidemic size for influenza B (FES*_B_*), fraction of QIV vaccinees, fraction of TIV vaccinees and the average social payoff (ASP). Clearly, the infection due to influenza B is dominating for the case, *β_A_* = *β_B_*. The fraction of TIV vaccinees is seen to prevail for certain regime as long as *C_T_* is below some threshold levels, however, QIV vaccinees are predominant whenever *C_Q_* and *C_T_* are comparable. Results from MAS approach show an overall similar tendency to that of mean-field approach. The MAS approach presumes 10 000 agents with *I_A_*(0) = 2 agents, *I_B_*(0) = 4 agents, *β_A_* = *β_B_* = 5.19957 × 10^−5^, *γ_A_* = *γ_B_* = 0.2, and takes ensemble average of 100 realizations. (Online version in colour.)

Figure 4 portrays several heatmaps depicting the final epidemic size for influenza A(B) virus-FES*_A_*(FES*_B_*), vaccination coverage for QIV(TIV) vaccine and the average social payoff-ASP (using equation (2.2)) in the steady state as a function of (*C_Q_*, *C_T_*) presuming an equal transmission rate for the both viruses (for the mean-field approach, we set *β_A_* = *β_B_* = 0.5 and *I_A_*(0) = 0.00001, *I_B_*(0) = 0.00002). It is worth mentioning that all heatmaps of our analyses presume the transition from the blue colour to the red colour as a good/better state to a bad/worse state of society in terms of infection, vaccination coverage and ASP, and vice versa. Clearly, infection due to influenza B virus predominates the other one that is akin to what we have observed in figure 2*a*,*b* for the case of equal transmission rates. As long as *C_T_* < *C_Q_* and *C_T_* is of lower than a certain threshold level, a broader regime in TIV heatmaps is covered by the blue colour (figure 2*a*-iv and *b*-iv); the counterpart of this region in QIV heatmaps is covered by the red colour, meaning that, because of lower cost, most individuals are interested in taking TIV vaccine until it bestows a considerable efficiency against B virus (that is of course below the efficacy of QIV vaccine). Despite a major portion taking TIV vaccine, it cannot fully suppress infection due to influenza B that can be clearly perceived from FES*_B_* heatmaps, which, in accordance, bestows a lower average payoff to society (figure 4*a*-v). However, the increase of effectiveness (TIV vaccine) against both viruses (if *e_Q_* = 0.8, *e_T_* = 0.6) would provide a better situation to society that can be observed from heatmaps in the second row. Remarkably, vaccination coverage for QIV vaccine seems to be dominant whenever both vaccination costs are comparable. Also, we can perceive the coexistence of both vaccinees in the borderline region between blue and red colours (yellowish regime enclosed by dotted lines in (*a*-iii) and (*a*-iv)).

Results obtained from the mean-field framework have been justified by a sequence of numerical simulations based on the MAS approach [18,40] presuming a complete graph as an underlying network (since we assume well-mixed population) with the population size, *N* = 10 000. The transmission rate parameters are chosen as *β_A_* = *β_B_* = 5.19957 × 10^−5^, which is estimated as the minimum transmission rate that surpasses the preset threshold final epidemic size of 0.9 without any vaccination [16]. The corresponding initial infections are *I_A_*(0) = 2 agents and *I_B_*(0) = 4 agents. Note that we estimate ensemble average for each 100 realizations. Generally, results obtained from the mean-field framework and MAS approach are showing overall the same tendency, albeit having subtle discrepancies in terms of colour scaling that comes from the fact that the MAS approach undertakes a finite population.

**Figure 5.**
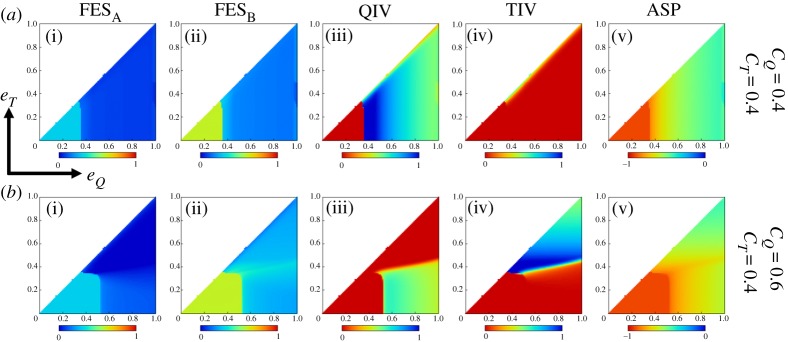
*e_Q_* versus *e_T_* (*e_T_* ≤ *e_Q_*) 2D-heatmaps with similar (upper panels) and different (lower panels) cost levels presuming mean-field framework with parameters choices, *β_A_* = *β_B_* = 0.5, *γ_A_* = *γ_B_* = 0.2, and initial conditions, *I_A_*(0) = 0.00001, *I_B_*(0) = 0.00002. Similar cost encourages people (with a higher degree) to commit QIV vaccine, consequently, showing sensitivity in the direction of *e_Q_*. However, in case of different costs (*C_Q_ *> *C_T_*), QIV vaccine is seen to favour over TIV vaccine when *e_Q_* ≥ 0.5 (approximately), on the other hand, TIV is favoured over QIV when *e_T_* ≥ 0.4 (approximately). The average social payoff for similar cost seems to be better than that of the different costs. Moreover, infection due to influenza A is lower than influenza B. (Online version in colour.)

Now let us focus on analysing similar phase diagrams by varying vaccine efficacies. To this aim, we generate several heatmaps as above as a function of (*e_Q_*, *e_T_*), 0 ≤ *e_T_* ≤ *e_Q_* ≤ 1, for the case of similar and different cost levels (figure 5). Obviously, similar cost for both vaccines would encourage people to choose QIV vaccine instead of TIV because the former one bestows better protection against both viruses. This situation illustrates why the sensitivity comes only along the direction of *e_Q_* (upper panels in figure 5). A lower degree of vaccine efficacy would not entice individuals to take vaccine (red region in figure 5*a*-iii); however, if *e_Q_* passes a threshold level, the fraction of QIV vaccinees mounts to the highest level (blue region in figure 5*a*-iii)). A further increase of *e_Q_* seems to suppress both infections significantly, which allows some people to avoid vaccination by free riding on herd immunity. This is why, after passing a transient regime having maximum vaccination coverage, the fraction of vaccinees decreases monotonically even with the increase of *e_Q_* (yellowish region in figure 5*a*-iii).

Nevertheless, if the cost for QIV is higher than TIV (lower panels in figure 5), then individuals' vaccination choice differs depending upon the degrees of *e_Q_* and *e_T_*. More specifically, under the current setting, QIV vaccine is favoured over TIV vaccine if *e_Q_* is above 50% (approximately) and *e_T_* is below 40% (approximately); on the other hand, if *e_T_* is above 40% (approximately), then TIV vaccine is preferred over QIV vaccine regardless of *e_Q_* (of course with the condition, *e_T_* ≤ *e_Q_*). Notably, in this case, the fraction of QIV vaccinees never reaches the maximum as the corresponding cost is higher; contrarily, TIV vaccinees are seen to reach the highest level for a mid-range of *e_T_*, although it starts decreasing with the further increase of *e_T_* that arises with the prevalence of free riders. Remarkably, the average payoff of society in case of different costs seems lower than that of the equal cost (see heatmaps for ASP in figure 5) as the former case imposes a higher financial burden to society.

#### (ii) Case II (*β_A_ *> *β_B_*)

**Figure 6.**
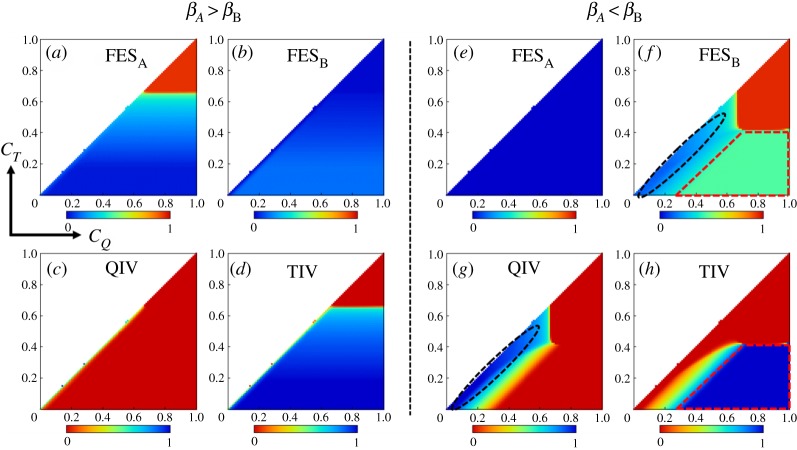
The heatmaps (*a*–*d*) show the fractions of infected individuals with influenza A and B viruses (FES*_A_*, FES*_B_*), fraction of QIV and TIV vaccinees as a function of *C_Q_* and *C_T_* (*C_T_* ≤ *C_Q_*), when the transmission rate for virus A is higher than B virus (*β_A_ *> *β_B_*;*β_A_* = 0.6, *β_B_* = 0.4). (*e*–*h*) The same objects for the case *β_A_* < *β_B_*; *β_A_* = 0.4, *β_B_* = 0.6. We choose, *e_Q_* = 0.6 and *e_T_* = 0.4 for both cases. We can perceive the infection dominance of A and B viruses according to the conditions *β_A_ *> *β_B_* and *β_A_* < *β_B_*. With the current setting, the case, *β_A_ *> *β_B_* implies the total predominance of TIV vaccinees (*e*,*f*). However, if *β_A_* < *β_B_*, then QIV vaccinees prevail whenever the cost difference is not so high and both costs are below 0.6 (approximately) but the prevalence of TIV vaccinees surges with the increase of *C_Q_* and the cost difference. The region where QIV is dominant shows more impact on disease suppression comparing to that of TIV (*f*,*g*,*h*). (Online version in colour.)

The case *β_A_ *> *β_B_* (*β_A_* = 0.6, *β_B_* = 0.4) leads to infection dominance of influenza A over B virus (figure 6*a*,*b*). This situation can be controlled by TIV vaccine alone as it targets two strains of A virus. Individuals would then mostly prefer TIV vaccine over QIV vaccine as the price of TIV is lower than the price of QIV (figure 6*c*,*d*). As a consequence, we perceive the sensitivity of choosing TIV vaccine only along the direction of *C_T_*.

#### (iii) Case III (*β_A_* < *β_B_*)

The predominance of influenza B virus over A virus creates a dilemma of choosing vaccine types. Individuals prefer QIV vaccine over TIV whenever both costs are comparable or the cost difference between QIV and TIV is not so high (region enclosed by black dotted lines in figure 6*g*); however, TIV vaccine seems mostly favourable in case of higher vaccination cost for QIV or higher cost difference between QIV and TIV vaccine (region enclosed by red dotted lines in figure 6*h*). Figure 6*g*,*h* also depicts the coexistence of both types of vaccinees in the yellowish transient regions. Furthermore, comparing the light blue region (enclosed with black dotted lines) with the greenish region (enclosed by red dotted lines) in figure 6*f*, it is observed that the QIV vaccine-dominant region imposes more effect on disease suppression compared to TIV vaccine.

#### (iv) *β_A_* versus *β_B_* phase plane

**Figure 7.**
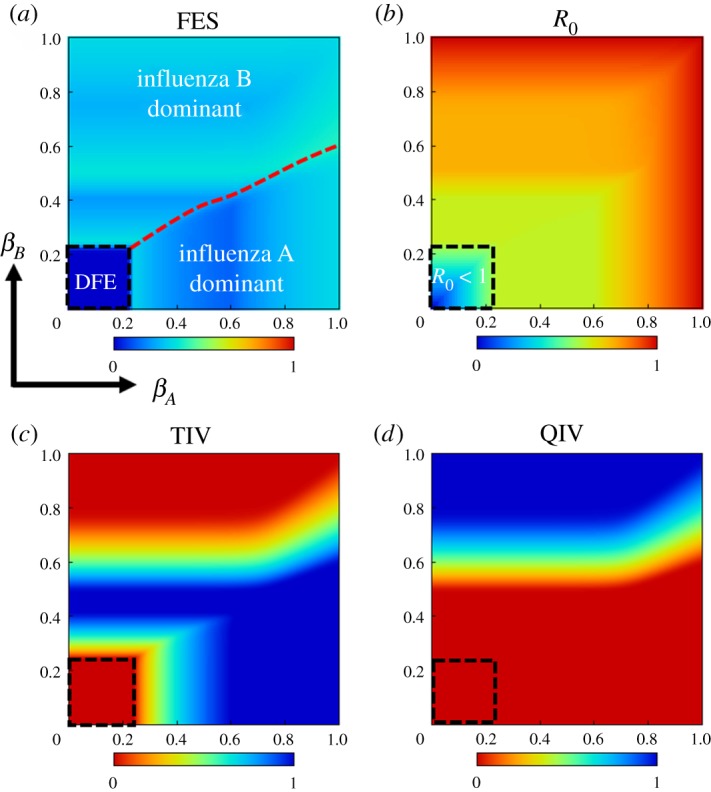
The effect of transmission rates (*β_A_*, *β_B_*) on disease spreading (*a*), the vaccine-dependent basic reproduction number-*R*_0_ (*b*), and the vaccination coverage of both vaccines (*c*,*d*). The deep blue region inside the box in (*a*) represents a DFE for both viruses, where *R*_0_ is below one (*b*), and accordingly, there is no vaccination coverage of any type inside the box. Outside of that box, influenza A(B) becomes prevalent with the increase of *β_A_*(*β_B_*). The red dotted line depicts the threshold level below(above) which influenza A(B) is dominant. TIV vaccinees predominate when *β_A_* gets larger and QIV vaccinees predominate when *β_B_* increases. The relevant parameters are set as *γ_A_* = *γ_B_* = 0.2; *e_Q_* = 0.6, *e_T_* = 0.4; *C_Q_* = 0.4, *C_T_* = 0.2. (Online version in colour.)

We intend to observe an overall impact of the transmission rates on disease propagation and vaccine uptake. To this aim, we draw heatmaps for total infection (FES) due to both viruses, the basic reproduction number (*R*_0_) and vaccination coverage for both vaccines (QIV, TIV), as a function of (*β_A_*, *β_B_*) (figure 7). The vaccine-dependent basic reproduction number *R*_0_ [54,55] is defined as $R_{0}=\max\{R_{0}^{A},R_{0}^{B}\},$ where $R_{0}^{A}$ and $R_{0}^{B}$ are the vaccine-dependent basic reproduction number for influenza A and B viruses, respectively. We derive $R_{0}^{A}$ and $R_{0}^{B}$ as

$$R_{0}^{A}=\frac{\beta_{A}}{\gamma_{A}}(S(0)+(1-e_{Q})(V_{Q}(0)+V_{T}(0)))$$

and

$$R_{0}^{B}=\frac{\beta_{B}}{\gamma_{B}}(S(0)+(1-e_{Q})V_{Q}(0)+(1-e_{T})V_{T}(0)).$$

Other relevant parameters are set as *γ_A_* = *γ_B_* = 0.2; *e_Q_* = 0.6, *e_T_* = 0.4; *C_Q_* = 0.4, *C_T_* = 0.2. Clearly, the increase of *β_A_*(*β_B_*) results in the upsurge of influenza A(B) virus. The deep blue regime inside the box illustrates the disease-free equilibrium (DFE) for both viruses, where *R*_0_ < 1 (figure 7*b*). Consequently, we observe no vaccination coverage inside that box (figure 7*c*,*d*). We perceive a red dotted line in FES-heatmap (figure 7*a*) below(above) which influenza A(B) predominates. As QIV vaccine provides better protection against B virus than TIV vaccine, we perceive the predominance of QIV vaccinees in case of higher *β_B_*; contrarily, TIV vaccine is favoured over QIV for higher *β_A_* because its price is lower than QIV.

### (d) Social efficiency deficit

Now we intend to explore how much the payoff attained at equilibrium falls short from the desired payoff or social optimum (SO) payoff. We name this payoff shortfall as the social efficiency deficit (SED) and define as

3.1 $$\text{SED},\;\delta =\text{AS}{\text{P}}^{\text{EQM}}-\text{AS}{\text{P}}^{\text{SO}},$$

where ASP^EQM^ and ASP^SO^ denote the average social payoff at equilibrium and social optimum, respectively. We use this parameter in explaining the degree of dilemma associated with each vaccine choice.

#### (i) Derivation of SED

As there are two vaccines available, individuals face a dual dilemma situation, choose QIV vaccine or TIV vaccine or none, depending upon their assessment on cost and effectiveness. These factors allow us to evaluate SED corresponding to both vaccines to ponder the impact of both types of vaccinations to society. The procedure given below can be followed in calculating SED for both vaccines. Suppose we wish to derive SED as a function of (*C_Q_*, *C_T_*). Note that it is also possible to express SED as a function of (*e_Q_*, *e_T_*).

Step (I) At first, we estimate ASP at equilibrium (EQM) as a function of (*C_Q_*, *C_T_*), for instance consider the heatmap for ASP given in figure 4*a*-v.

**Figure 8.**
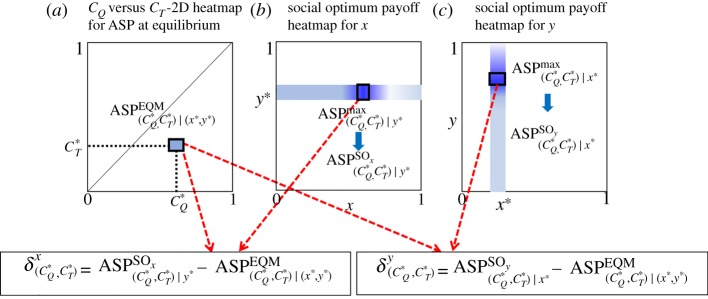
The schematic of SED derivation. At first, we choose a point ($C_{Q}^{*}$,$C_{T}^{*}$) on the heatmap (*C_Q_* versus *C_T_*) illustrating the average social payoff (ASP) at equilibrium (EQM) (*a*), in which there is a corresponding point, (x*, y*), i.e. a corresponding pair of vaccinees (QIV, TIV). With fixed ($C_{Q}^{*}$,$C_{T}^{*}$) and y*, we vary *x* (0 ≤ *x* ≤ 1) and estimate the maximum average payoff, which we term as social optimum (SO) payoff for *x* at ($C_{Q}^{*}$,$C_{T}^{*}$) and y*, i.e. ${\text{ASP}}_{\left(C_{Q}^{*},C_{T}^{*}\text{|}y^{*}\right)}^{\text{S}{\text{O}}_{x}}$ (*b*). After that we derive SED for *x* at ($C_{Q}^{*},C_{T}^{*}$) by subtracting ASP at equilibrium from the payoff at social optimum. We follow the same procedure to derive SED for *y* using (*a*) and (*c*). (Online version in colour.)

Step (II) Each point on this heatmap corresponds to a pair, $\left(C_{Q}^{*},C_{T}^{*}\right)$. Also, there is a corresponding pair (x*, y*), i.e. vaccination coverage for each vaccine, for each pair $\left(C_{Q}^{*},C_{T}^{*}\right)$. Now choosing y*(x*) as fixed and varying *x*(*y*) from 0 up to 1, we estimate the maximum average payoff (using formula (2.2)), what we call a social optimum payoff for *x*(*y*) at the point $\left(C_{Q}^{*},C_{T}^{*}\right)$ (figure 8*a*–*c*).

Step (III) Next, we derive SED for *x*(*y*) at the point $\left(C_{Q}^{*},C_{T}^{*}\right)$ by simply subtracting ASP attained in step I from the social optimum payoff attained in step II and denote it by $\delta_{\left(C_{Q}^{*},C_{T}^{*}\right)}^{x}\left(\delta_{\left(C_{Q}^{*},C_{T}^{*}\right)}^{y}\right)$, that is

$$\delta_{\left(C_{Q}^{*},C_{T}^{*}\right)}^{x}={\text{ASP}}_{(C_{Q}^{*},C_{T}^{*})|y^{*}}^{\text{S}{\text{O}}_{x}}-{\text{ASP}}_{\left(C_{Q}^{*},C_{T}^{*})|(x^{*},y^{*}\right)}^{\text{EQM}}$$

and

$$\delta_{\left(C_{Q}^{*},C_{T}^{*}\right)}^{y}={\text{ASP}}_{(C_{Q}^{*},C_{T}^{*})|x^{*}}^{\text{S}{\text{O}}_{y}}-{\text{ASP}}_{\left(C_{Q}^{*},C_{T}^{*})|(x^{*},y^{*}\right)}^{\text{EQM}}.$$

**Figure 9.**
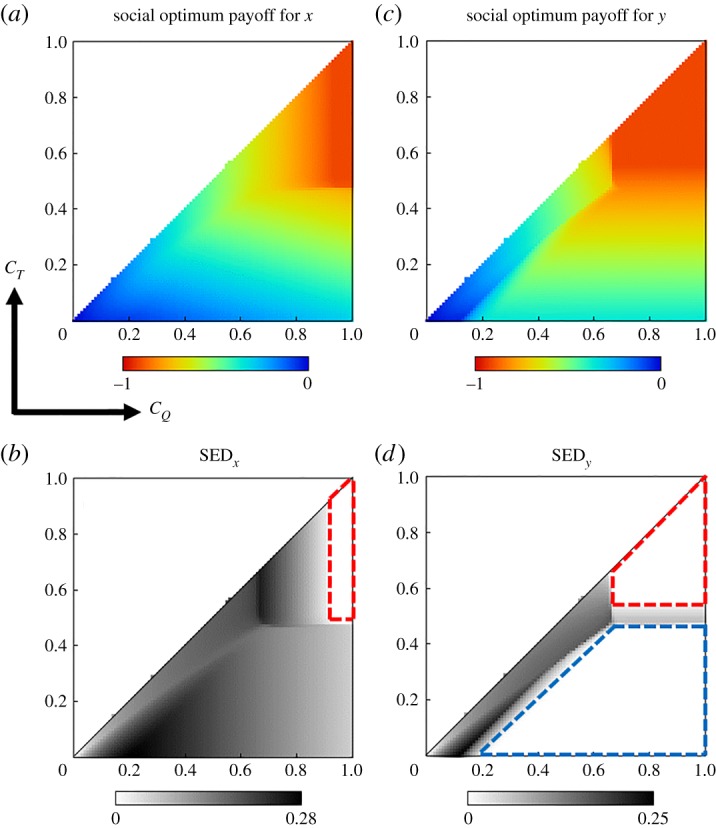
Representation of social optimum payoff (*a*,*b*) and SED (*c*,*d*) for *x* (fraction of QIV vaccinees) and *y* (fraction of TIV vaccinees) as a function of (*C_Q_*, *C_T_*). SED heatmaps are attained by subtracting the ASP at equilibrium (figure 4*a-*v) from the social optimum (SO) payoffs for *x* and *y* (*a*,*b*). The relevant parameters are chosen as: *e_Q_* = 0.6, *e_T_* = 0.4, *β_A_* = *β_B_* = 0.5 and *γ_A_* = *γ_B_* = 0.2. Referring to figure 4*a*-i–*a*-iv, these parameter choices depict the prevalence of influenza B virus and lower vaccination coverage for QIV vaccine (especially when the cost difference is high). The whiteout regions in (*c*,*d*) depict that there is no gap between SO payoff (for *x* or *y*) and ASP at equilibrium. More areas in SED-heatmap for *x* appears to have darker regime than that of the case for *y*, which indicates the higher gap between SO payoff for *x* and ASP at equilibrium. (Online version in colour.)

Figure 8 schematically explains the whole procedure. Now let us explain the heatmaps for SED that are generated following the above procedure. As an archetype, we choose the case illustrated in the upper panel of figure 4, where we assumed *β_A_* = *β_B_* = 0.5 and *e_Q_* = 0.6, *e_T_* = 0.4. In that case, we observed that most of the people are inclined to choose TIV vaccine whenever the cost difference between *C_Q_* and *C_T_* gets higher but individuals prefer QIV if the cost difference becomes lower. It is conceivable that the overall desired payoff or social optimum payoff for QIV vaccinees (*x*) would be relatively higher than the TIV vaccinees (*y*), which is appeared in figure 9*a*,*b*, but in this case, the vaccination coverage for QIV seems much lower than TIV (figure 4*a*-iii–*a*-iv), which indicates that the dilemma of choosing QIV vaccine is higher than the other one. Obviously, this comes from the fact of higher cost associated with QIV vaccine. This factor results in less contribution of QIV vaccine to ASP (figure 4*a*-v). As a result, we perceive relatively higher payoff-gap between social optimum payoff for *x* and ASP (at equilibrium); hence, more areas of the heatmap for SED*_x_* are seemed dark compared to its counterpart (SED*_y_*). The whiteout region in heatmaps (figure 9*c*,*d*) depicts the scenario with no SED, that is there is no gap between social optimum payoff and ASP. There are two white regions in the SED*_y_*-heatmap (figure 9*d*), which either come from 100% TIV vaccination coverage (region enclosed by blue dotted lines) resulting from lower *C_T_* and higher *C_Q_* (blue region in figure 4*a*-iii), or from the scenario when everyone becomes a free rider (region enclosed by red dotted lines in figure 9*b*,*d*). The latter situation can arise for higher vaccination cost or for very low vaccine effectiveness. In this case, avoiding any type of vaccination is the best strategy (desired strategy), which agrees with the D(unvaccinated)-dominant equilibrium (figure 4*a*-iii,*a*-iv). As a result, the payoff at this equilibrium also agrees with the social optimum payoff; hence no SED or no dilemma arises in this case.

## 4. Conclusion

In this paper, we consider a simultaneous spreading of two influenza viruses (A and B) coupled with two types of vaccinations. The QIV vaccine contains one extra strain of influenza B virus and hence provides better protection compared to the TIV vaccine. However, the cost of the QIV vaccine is higher than TIV, which may create a dilemma for individuals’ vaccination choice. Here we design a mean-field vaccination game model in an infinite and well-mixed population to explain people's vaccination dilemma, entangling an epidemic spreading process of influenza with the coevolution of two types of vaccinations. Our evolutionary framework assumes three strategies—choose QIV vaccine, TIV vaccine or none—that evolve at the end of each season by imitating strategies based on the previous season's experience. We perform a series of numerical simulations by varying vaccination cost, vaccine effectiveness, transmission rates, etc., to depict different scenarios. Our results show that individuals are more inclined to take QIV vaccine whenever both vaccination costs are comparable; however, they prefer TIV vaccine if the cost difference gets higher. Notably, the framework has been validated by the so-called MAS approach.

Generally, people's vaccination choice is influenced by the costs as well as vaccine efficacies; however, our results show that individuals' vaccination options (QIV or TIV) are more affected by the cost difference of both vaccines as long as the vaccine with the lower price is able to bestow a considerable level of efficiency, especially against B virus. Another interesting result concerns the variation of transmission rates for both viruses and its resulting effect on disease propagation and vaccination choice. The higher transmission rate of influenza A virus leads to a maximum coverage for TIV vaccine since it possesses the same degree of effectiveness against influenza A virus as that of QIV vaccine but with a lower cost; contrarily, the higher transmission rate of influenza B virus results in almost complete dominance of QIV vaccinees that is quite conceivable.

Our analysis also incorporates how much the overall payoff or the average social payoff (ASP) attained by the process of voluntary vaccination (at equilibrium) falls short from the desired payoff or social optimum payoff, what we have termed as social efficiency deficit (SED)—the payoff gap between social optimum and equilibrium. This parameter also helps us elucidate the degree of dilemma associated with each vaccination choice. It appears that for the case of equal transmission rates, people face a bigger dilemma to choose QIV vaccine rather than TIV that arises from the fact of the higher vaccination cost for QIV even though it confers relatively better protection.

In sum, this work endeavours to explore individuals’ dilemma on choosing influenza vaccines through the lens of a vaccination game, where the disease spreading process is modelled by a simple SIR-like model coupled with two types of vaccinations. Our approach does not consider any strain-specific infection, rather it relies on virus-specific infection (influenza A and B) with long-term cross-immunity. It would be interesting to explore strain-specific infection, where an individual infected with a particular strain can have short-term or long-term cross-immunity against the other one (e.g. [49]). The inclusion of age structure (for instance [49,56]) in the current framework could be another aspect to explore. The influenza epidemic varies from one country to another country, thus it would be also interesting to explore the outlined framework for a specific country with proper estimation of relevant parameters [56,57]. In addition to a voluntary vaccination campaign, subsidies [14,58] provided by the government (especially for QIV vaccine) can enhance vaccination coverage to prevent disease-outbreak more efficiently. Therefore, the current framework with a subsidy provision could be another avenue to explore.

In the present study, we have presumed a well-mixed situation. The next step for the current framework may be the inclusion of an underlying network. The focal point of our present model is plural vaccinations having different capabilities and costs for plural viruses of the same disease. Such a specific but quite realistic situation would be significantly influenced by a spatial structure connecting individuals. The well-mixed situation can be said, in a sense, to be an ideal assumption because the social information helping individuals to decide for vaccination is quickly shared and can also be said to be an extreme assumption because disease spreading occurs quickly vis-à-vis the case with the spatial structure. The time delay between information and disease spreading on the underlying network might be more significant for the model with plural vaccinations and plural viruses (or plural strains) than the conventional setting with a single vaccination and a single strain.

## Supplementary Material

Source code (c++)

## Data accessibility

All data of this research work have been generated from a series of numerical simulations. The source code (c++) of our numerical simulation has been uploaded as electronic supplementary material in the ‘File Upload’ section.
