# Supplementary material for: Interplay between cost and effectiveness in influenza vaccine uptake: a vaccination game approach
Source: Proc Math Phys Eng Sci. 2019 Dec 18;475(2232):20190608. doi: 10.1098/rspa.2019.0608 (PMC6936611; doi:10.1098/rspa.2019.0608)
Supplement: Source code (c++) [file rspa20190608supp1.pdf]

Source code (c++) of the simulation

```
// This code is written by Md. Rajib Arefin
// Date: 20/05/2019
// This c++ code is written to simulate a mean-field approach in vaccination game to explore…
// …individuals vaccination dilemma in influenza vaccine uptake depending upon cost and efficacy
// This source code can serve as the base program for generating data for Figs. 4 - 7 and 9
// Specifically, this code generates data for all C_Q vs C_T heatmaps in Fig. 4. with fixed e_Q,
e_T_B
// By changing parameter settings, all other heatmaps can be generated

/////////////////////////////////////////////////////////////////////////////////////////
#include <iostream>
#include <fstream>
#include <stdio.h>
#include <math.h>
#include <vector>
#include <list>
#include <string.h>
#include <stdlib.h>
#include <ctype.h>
#include <sstream>
#include <string>
#include <cstdio>
#include <time.h>
#include <iomanip>
using namespace std;
using std::setw;

int main()
{
# define times 300
# define GENERATION 1000
# define cost 100
# define quality 100
# define K 0.1 // sensitivity constant used in Fermi rule
// defining transmission rate and recovery rate parameters
# define B1 0.5 //
# define B2 0.5
# define G1 0.2
# define G2 0.2
// creating csv file for storing several results coming from the simulation
       string file1 = "total_infected_fraction.csv";
```

```cpp
ofstream Data1(file1);
Data1 << "C_Q,C_T,R" << endl;

string file2 = "VC_Q.csv";  // vaccination coverage (QIV)
ofstream Data2(file2);
Data2 << "C_Q,C_T,V_Q" << endl;

string file3 = "VC_T.csv";   // vaccination coverage (TIV)
ofstream Data3(file3);
Data3 << "C_Q,C_T,V_T" << endl;

string file4 = "ASP.csv"; // average social payoff
ofstream Data4(file4);
Data4 << "C_Q,C_T,asp" << endl;

string file5 = "total vaccination coverage.csv";
ofstream Data5(file5);
Data5<< "C_Q,C_T,vaccination_coverage" << endl;

string file6 = "fes_A.csv"; // infection by flu A virus
ofstream Data6(file6);
Data6 << "C_Q,C_T,fesA" << endl;

string file7 = "fes_B.csv";  // infection by flu B virus
ofstream Data7(file7);
Data7 << "C_Q,C_T,fesB" << endl;

// Declaring several variables for epidemic dynamics
double S[times + 1], VQ[times + 1], VT[times + 1], IA[times + 1], IB[times + 1],
      R[times + 1];

// several variables for flux calculations
double S1[times + 1], S2[times + 1],V1[times + 1],V2[times + 1],FA[times + 1],FB[times + 1];
double IfaVQ[times + 1], IfbVQ[times + 1], IfaVT[times + 1], IfbVT[times + 1];//several flux
double x[GENERATION + 1]; // fraction of QIV vaccinees
double y[GENERATION + 1]; // fraction of TIV vaccinees
double asp[GENERATION + 1]; // average social payoff
double C_Q, C_T, e_Q, e_T_A, e_T_B;// cost and effectiveness parameters
double HVQ, IVQ, SFR, FFRa, HVT, IVT, FFRb; // outcome of the epidemic (several fractions)
// several transitional probabilities
double phVQ_NV, phVQ_VT, piaVQ_NV, pibVQ_NV, pibVQ_VT, phVT_NV, piaVT_NV, pibVT_NV, piaVT_VQ,
pibVT_VQ, piaVQ_VT;
```

```
        double psfr_VQ, psfr_VT, pffra_VQ, pffra_VT, pffrb_VQ, pffrb_VT, VC_Q, VC_T, piVQ_VT,
        phVT_VQ, NV;
        double pi_Q, pi_T, pi_NV; // average payoff for QIV, TIV, and non-vaccinees

        x[0] = 1.0 / 3.0; y[0] = 1.0 / 3.0;  // initial value of QIV and TIV vaccinees
        e_Q = 0.6; e_T_B = 0.4;
        for (int cs = 0; cs < cost + 1; cs++)    // Calculating cost for QIV vaccine (loop 1)
        {
                C_Q = cs / 100.0;
                for (int cc = 0; cc < C_Q * 100 + 1; cc++) //Calculating cost for TIV vaccine
(C_T<=C_Q) (loop 2)
                {
                        C_T = cc / 100.0;
                        for (int j = 0; j < GENERATION; j++)// number of seasons/episode loop (loop 3)
                        {
                                IA[0] = 0.00001;IB[0] = 0.00002; R[0] = 0.0;// initializing infected
fractions and recovered fractions
                                VQ[0] = x[j]; VT[0] = y[j]; IfbVT[0] = 0.0; IfaVT[0] = 0.0; IfbVQ[0] =
0.0; IfaVQ[0] = 0.0;
                                S[0] = 1.0 - x[j] - y[j] - IA[0] - IB[0]; FA[0] = 0.0; FB[0] = 0.0;
                                S1[0] = 0.0; S2[0] = 0.0; V1[0] = 0.0; V2[0] = 0.0;
                                e_T_A = e_Q;
                               for (int i = 0; i < times; i++){//SIR dynamics with vaccinations (loop 4)
                                S[i + 1] = S[i] - S[i] * (B1 * IA[i] + B2 * IB[i]);


                                VQ[i + 1] = VQ[i] - (VQ[i] - e_Q * VQ[0]) * B1 * IA[i] - (VQ[i] - e_Q *
VQ[0]) * B2 * IB[i];


                                VT[i + 1] = VT[i] - (VT[i] - e_T_A * VT[0]) * B1 * IA[i] - (VT[i] -
e_T_B * VT[0])* B2 * IB[i];


                                IA[i + 1] = IA[i] + B1 * IA[i] * (S[i] + VQ[i] - e_Q * VQ[0]) + (VT[i] -
e_T_A * VT[0])* B1 * IA[i] - G1 * IA[i];


                                IB[i + 1] = IB[i] + B2 * IB[i] * (S[i] + VT[i] - e_T_B * VT[0]) + (VQ[i]
- e_Q * VQ[0]) * B2 * IB[i] - G2 * IB[i];


                                R[i + 1] = R[i] + G1 * IA[i] + G2 * IB[i];


                                // calculating several fluxes
                                S1[i + 1] = S1[i] + S[i] * B1 * IA[i]; // calculating flux for failure
free rider for A virus
```

```
                                S2[i + 1] = S2[i] + S[i] * B2 * IB[i];   // calculating flux for failure
free rider for B virus
                                        // calculating flux for infected but vaccinated people (QIV)
                                V1[i + 1] = V1[i] + (VQ[i] - e_Q * VQ[0]) * B1 * IA[i] + (VQ[i] - e_Q *
VQ[0]) * B2 * IB[i];
                                        // calculating flux for infected but vaccinated people (TIV)
                                V2[i + 1] = V2[i] + (VT[i] - e_T_B * VT[0]) * B2 * IB[i] + (VT[i] -
e_T_A * VT[0])* B1 * IA[i];

                                        //total infection with flu A
                                FA[i + 1] = FA[i] + B1 * IA[i] * (S[i] + VQ[i] - e_Q * VQ[0]) + (VT[i] -
e_T_A * VT[0])* B1 * IA[i];
                                        //total infection with flu B
                                FB[i + 1] = FB[i] + B2 * IB[i] * (S[i] + VT[i] - e_T_B * VT[0]) + B2 *
(VQ[i] - e_Q * VQ[0]) * IB[i];
                                // flux from VQ to A virus
                                IfaVQ[i + 1] = IfaVQ[i] + (VQ[i] - e_Q * VQ[0]) * B1 * IA[i];
                                // flux from VQ to B virus
                                IfbVQ[i + 1] = IfbVQ[i] + (VQ[i] - e_Q * VQ[0]) * B2 * IB[i];
                                // flux from VT to A virus
                                IfaVT[i + 1] = IfaVT[i] + (VT[i] - e_T_A * VT[0])* B1 * IA[i];
                                // flux from VT to B virus
                                IfbVT[i + 1] = IfbVT[i] + (VT[i] - e_T_B * VT[0]) * B2 * IB[i];
                                } // loop 4 ends here
                                 // outcome of the epidemics
                                HVQ = VQ[times];HVT = VT[times]; // healthy QIV and TIV vaccinees
                                // QIV and TIV vaccinees but infected
                                IVQ = IfaVQ[times] + IfbVQ[times]; IVT = IfaVT[times] + IfbVT[times];
                                FFRa = S1[times]; FFRb = S2[times];      // failed free riders
                                SFR = S[times]; // successful free riders
                                pi_Q = (HVQ*(-C_Q) + IVQ * (-C_Q - 1.0)) / (HVQ + IVQ); // average
payoff for QIV vaccinees
                                pi_T = (HVT*(-C_T) + IVT * (-C_T - 1.0)) / (HVT + IVT); // average
payoff for TIV vaccinees
                                pi_NV = (SFR*(0.0) + (FFRa + FFRb)*(-1.0)) / (SFR + FFRa + FFRb); //
average payoff for non-vaccinees
                                asp[j] = -C_Q * HVQ - C_T * HVT - (C_Q + 1)*IVQ - (C_T + 1)* IVT - (FFRa
+ FFRb);

                                // transition probabilities (Fermi rule)
                                phVQ_NV = 1.0 / (1 + exp(-(pi_NV - (-C_Q)) / K)); // P(healthy
vaccinators(QIV) copying non-vaccinators' strategy)
```

```
                        phVQ_VT = 1.0 / (1 + exp(-(pi_T - (-C_Q)) / K));
                        piaVQ_NV = 1.0 / (1 + exp(-(pi_NV - (-C_Q - 1)) / K));
                        pibVQ_NV = 1.0 / (1 + exp(-(pi_NV - (-C_Q - 1)) / K));
                        piaVQ_VT = 1.0 / (1 + exp(-(pi_T - (-C_Q - 1)) / K));
                        pibVQ_VT = 1.0 / (1 + exp(-(pi_T - (-C_Q - 1)) / K));
                        phVT_NV = 1.0 / (1 + exp(-(pi_NV - (-C_T)) / K));
                        phVT_VQ = 1.0 / (1 + exp(-(pi_Q - (-C_T)) / K));
                        piaVT_NV = 1.0 / (1 + exp(-(pi_NV - (-C_T - 1)) / K));
                        pibVT_NV = 1.0 / (1 + exp(-(pi_NV - (-C_T - 1)) / K));
                        piaVT_VQ = 1.0 / (1 + exp(-(pi_Q - (-C_T - 1)) / K));
                        pibVT_VQ = 1.0 / (1 + exp(-(pi_Q - (-C_T - 1)) / K));
                        psfr_VQ = 1.0 / (1 + exp(-(pi_Q - 0) / K));
                        psfr_VT = 1.0 / (1 + exp(-(pi_T - 0) / K));
                        pffra_VQ = 1.0 / (1 + exp(-(pi_Q - (-1.0)) / K));
                        pffra_VT = 1.0 / (1 + exp(-(pi_T - (-1.0)) / K));
                        pffrb_VQ = 1.0 / (1 + exp(-(pi_Q - (-1.0)) / K));
                        pffrb_VT = 1.0 / (1 + exp(-(pi_T - (-1.0)) / K));

                        // total fraction of QIV, TIV and non-vaccinees
                        VC_Q = HVQ + IVQ; VC_T = HVT + IVT; NV = SFR + FFRa + FFRb;

                        // evolutionary dynamics of QIV vaccinees
                        x[j + 1] = x[j] - HVQ * NV*phVQ_NV - IfaVQ[times] * NV*piaVQ_NV -
IfbVQ[times] * NV*pibVQ_NV - HVQ * VC_T*phVQ_VT -IfaVQ[times] * VC_T*piaVQ_VT - IfbVQ[times] *
VC_T*pibVQ_VT + SFR * VC_Q*psfr_VQ + FFRa * VC_Q*pffra_VQ +FFRb * VC_Q*pffrb_VQ + HVT * VC_Q*phVT_VQ
+ IfaVT[times] * VC_Q*piaVT_VQ + IfbVT[times] * VC_Q*pibVT_VQ;
                        // evolutionary dynamics of TIV vaccinees
                        y[j + 1] = y[j] - HVT * NV*phVT_NV - IfaVT[times] * NV*piaVT_NV -
IfbVT[times] * NV*pibVT_NV - HVT * VC_Q*phVT_VQ -IfaVT[times] * VC_Q*piaVT_VQ - IfbVT[times] *
VC_Q*pibVT_VQ + SFR * VC_T*psfr_VT + FFRa * VC_T*pffra_VT + FFRb * VC_T*pffrb_VT + HVQ *
VC_T*phVQ_VT + IfaVQ[times] * VC_T*piaVQ_VT + IfbVQ[times] * VC_T*pibVQ_VT;

                } // loop 3 ends here
                // saving data in csv file
                Data1 << C_Q << "," << C_T << "," << R[times] << endl;
                Data2 << C_Q << "," << C_T << "," << x[GENERATION] << endl;
                Data3 << C_Q << "," << C_T << "," << y[GENERATION] << endl;
                Data4 << C_Q << "," << C_T << "," << asp[GENERATION - 1] << endl;
                Data5 << C_Q << "," << C_T << "," << x[GENERATION] + y[GENERATION] << endl;
                Data6 << C_Q << "," << C_T << "," << FA[times] << endl;
                Data7 << C_Q << "," << C_T << "," << FB[times] << endl;
        } // loop 2 ends here
```

```
        } // loop 1 ends here
        Data1.close();
        Data2.close();
        Data3.close();
        Data4.close();
        Data6.close();
        Data7.close();
} // program ends here
```
